# 

**Published:** 1919-07-12

**Authors:** 


### Medical Protection.

The annual report of the Council of London and Counties Medical Protection Society appears once more in the abbreviated form dictated by war conditions. The financial security of the Society has been further strengthened during the year (1918) under review, as is evident from the accounts presented, which show surpluses for the year of about £200 on the income and expenditure account; and of about £2,800 on the Reserve and Insurance Fund accounts. The annual meeting took place on July 2, at the Society's offices, 32 Craven Street, Strand.

Mr. Arthur Earle, of Liverpool, among other charitable bequests left £1,000 each to the following Liverpool institutions: David Lewis Hospital, the Royal Infirmary, the Royal Southern Hospital, the Infirmary for Children, and St. Paul's Eye and Ear Hospital.

### Gifts and Bequests.

MISS EMILY THOMAS, of Bayswater, bequeathed £500 to the Cancer Charity of the Middlesex Hospital and £300 to the Royal Dental Hospital, Leicester Square.

UNDER the will of Lady Weld, of Llwynarthan, Newport, £5,000 is provided for a recreation hall at the South Wales Sanatorium of the King Edward VII. Welsh National Memorial, £2,000 is left to the Nation's Fund for Nurses, £1,050 to the King Edward VII. Hospital, Cardiff, and £1,050 to the Prince of Wales's Hospital for Limbless Soldiers, Cardiff.

ONE THOUSAND POUNDS has been bequeathed to the Central Nose, Ear and Throat Hospital by Mrs. Mary Lucinda Browne, of Matlock.

### MATRONS' AND SISTERS' APPOINTMENTS.

Stamford, Rutland, and General Infirmary, Stamford.—Miss Ethel Sowden has been appointed sister of the diphtheria block. She was trained at the Kendray Fever Hospital, Barnsley, has been staff nurse at the Earsdon Hospital and the Birmingham City Hospital, sister at Watford Isolation Hospital, and night sister at the Edmonton and Enfield Hospital.

Queen's Hospital, Birmingham. — Miss E. Bullivant, A.R.R.C., has been appointed matron. She was trained at the above hospital, at which she has since been ward, senior massage and home sister, and assistant matron. Miss Bullivant holds the certificates of the Incorporated Society of Trained Masseuses and of the Central Midwives Board.

King Hill Institution, West Malling.—Miss Amelia A. Alder has been appointed head nurse. She was trained at Westminster Infirmary, has been superintendent nurse at the Uxbridge Union Infirmary, and has been night sister under the Metropolitan Asylums Board.

Eccles and Patricroft Hospital, near Manchester.—Miss M. A. Allen and Miss A. Donohue have been appointed sisters. Miss Allen was trained at Huddersfield Royal Infirmary, has been on the private nursing staff of Preston Royal Infirmary, sister at St. Vincent's Cripples' Home, Pinner, and at Warrington General Infirmary, and sister in Queen Alexandra's Imperial Military Nursing Service Reserve, serving at Dublin and in Mesopotamia. Miss Donohue was trained at Blackburn General Infirmary, and has also had fever training. She has since been doing private nursing, and has served with Queen Alexandra's Imperial Military Nursing Service Reserve.

Isolation Hospital and Sanatorium, Chadwell, Essex.—Miss Helena McLoughlin has been appointed assistant matron. She was trained at the Swindon Isolation Hospital, and has held appointments at the Royal Hospital, Portsmouth; the Colchester Military Hospital; and the Cairo Military Hospital.

Isolation Hospital, Shepton Mallet.—Mrs. Mary Bristow has been appointed nurse-matron. She was trained at the Bristol General Hospital, has been staff nurse at the Military Hospital, Shepton Mallet, and sister at the County Sanatorium, Shepton Mallet.

Monkwearmouth and Southwick Hospital, Sunderland.—Miss Margaret K. Jones has been appointed night sister. She was trained at Leicester Royal Infirmary, and during the fourth year of training has taken sister's duties.

Lambert Memorial Hospital, Thirsk, Yorks.—Miss Ellen Goodwill has been appointed nurse-matron. She was trained at the North Riding Infirmary, Middlesbrough, where she has since been theatre sister and night sister, and has been temporary matron at the Cottage Hospital, Whitby.

General Hospital, Newry, County Down.—Miss Mary Mulligan has been appointed sister. She was trained at the Mater Misericordiæ Hospital, Dublin, and has since been doing private nursing.

St. John's Hospital for Diseases of the Skin, 262 Uxbridge Road, W.—Miss Isabella E. B. Moore has been appointed matron. She was trained at Glasgow Royal Infirmary, where she has since been assistant housekeeper and housekeeper, and has been housekeeper at the 3rd and 4th Scottish General Hospitals, Stobhill, Glasgow. Miss Moore has served as staff nurse in the Territorial Force Nursing Service.

York County Hospital.—Miss Florence Moxon has been appointed sister. She was trained at the Royal Infirmary, Sheffield, has been theatre sister at the County Hospital, Guildford, at the Racecourse Hospital, Cheltenham, and has served in Italy in Queen Alexandra's Imperial Military Nursing Service Reserve.

Ellen Gonner Convalescent Home for Children, Hoylake, Cheshire.—Miss A. Crossley Gregson has been appointed sister. She was trained at Warrington Infirmary and at Bolton Fever Hospital, has been charge nurse at Chorley Union Infirmary, head nurse at Swinton Schools, night superintendent at the Union Infirmary, Stockport, and has done temporary district nursing.

Isolation Hospital, Gillingham, Kent.—Miss Ethel A. Abbs has been appointed matron. She was trained at St. Mary's Hospital, London, and at Plaistow Fever Hospital, at which latter institution she has since been ward sister, night superintendent, and acting assistant matron. Miss Abbs has also been assistant matron at the National Hospital for the Paralysed and Epileptic, Bloomsbury, and has helped in the organisation of a hospital for French soldiers in France.

King Edward VII.'s Convalescent Home for Officers, Osborne.—Miss Annie Elizabeth Dunton has been appointed sister. She was trained at the London Hospital, was afterwards on the London Hospital private nursing staff, and later nurse-matron at the London Hospital Convalescent Homes for Children, Pevensey Bay. She has since been night superintendent at the Exeter War Hospitals No. V. and No. I, and ward sister at the Norfolk War Hospital, Thorpe.

### THE COLLEGE OF NURSING (Limited by Guarantee).

### THE LONDON CENTRE.

Mrs. M. L. Carter, Acting Hon. Secretary to London Centre, asks us to point out that only members of the College are eligible for membership of the London or any other Local Centre. Members of the College wishing to join the London Centre should address their applications, giving their registration number, to the Hon. Secretary, the London Centre Club, 7 Henrietta Street, Cavendish Square, W. 1.

Nurses applying for membership of the College should address their applications to the Registrar, the College of Nursing, 7 Henrietta Street, Cavendish Square, W. 1.

No meeting of the Centre will be held during August and September, but the club rooms at 7 Henrietta Street, W. 1, will remain open for the use of members. The winter session will open early in October, particulars of which will be published later.

### ABERDEEN CENTRE.

A very interesting visit was enjoyed by the members who paid a visit to the "Hatchery" on the last Thursday in June. A description of the different kinds of fish and the methods of rearing them was given by Miss Gilbert, who had no difficulty in securing the rapt attention of her audience. Tea was served in the garden overlooking the Bay of Nigg, and the afternoon concluded with a walk round the cliffs.

### LIVERPOOL CENTRE.

We are asked to state that at a members' meeting, held after the committee meeting at the Royal Infirmary on Friday, July 4, the proposed "Chair of Nursing" to be supported by the College of Nursing was discussed. It was unanimously agreed by all members present to support the project.

The position of Registration was explained to the meeting by Miss Cummins, whose remarks were followed with keen interest.

